# Comment on “Pooled analysis of Xpert bladder cancer based on the 5 mRNAs for rapid diagnosis of bladder carcinoma”

**DOI:** 10.1186/s12957-021-02359-3

**Published:** 2021-08-11

**Authors:** Gopal Sharma

**Affiliations:** Department of Urology, PGIMER, Chandigarh, 160012 India

**Keywords:** Bladder cancer, NMIBC, Xpert BC

## Abstract

This article is a commentary on an article published in *World Journal of Surgical Oncology* by Liu et al. With this study, authors evaluated diagnostic accuracy of Xpert bladder cancer monitor in detecting recurrences. With this article, we highlight the strengths and limitations of the study.

Sir,

I read an article published by Liu et al. [1] in the February edition of World Journal of Surgical Oncology. I read this article with keen interest and would extend my best wishes to the study authors. This study aimed to “reviewing the performance of Xpert BC in the follow-up of bladder cancer and evaluating the role of Xpert BC in detecting NMIBC recurrence in the round”. The study has been well performed in my opinion; however there are certain issues which I would like to highlight.

1. I have reservations over inclusion of study by Valenberg et al. [2], as this study tested accuracy of Xpert bladder cancer (BC) in patients who presented with hematuria. Hence still study does not meet the primary aim of this study, which was to detect diagnostic accuracy of Xpert BC in patients with bladder cancer on follow-up.
2. Study authors have pooled overall data of study by Wallace et al. [3] that included patients with both known case of bladder cancers (on surveillance) and patients who presented with hematuria. Thus, in my opinion separate data for patients with BC on surveillance should be extracted and reanalyzed.
3. Lastly, use of different gold standard techniques by different studies is yet another limitation of this study which needs to be highlighted. Subgroup analysis according to the gold standard modality could also be performed.

To conclude, the results of this study need to interpret with caution due to abovementioned limitations and if possible needs revision for above mentioned points as they have potential to change the overall results.

## Data Availability

All data generated or analyzed during this study are included in this published article.
